# Depression Symptoms among Family Members of Nyaope Users in the City of Tshwane, South Africa

**DOI:** 10.3390/ijerph19074097

**Published:** 2022-03-30

**Authors:** Maphuti Carol Madiga, Kebogile Mokwena

**Affiliations:** 1 National Research Foundation, Pretoria 0001, South Africa; 2 Substance Abuse and Population Mental Health, Sefako Makgatho Health Sciences University, Pretoria 0204, South Africa; kebogile.mokwena@smu.ac.za

**Keywords:** depression symptoms, South Africa, patient health questionnaire-9 (PHQ-9), nyaope, family

## Abstract

Substance abuse brings major negative social and health impacts in South Africa. Nyaope, a cocktail drug commonly used in the Tshwane townships, has been well documented to be highly addictive and very difficult to quit. The resultant difficulties include financial, social, and mental, specifically depression and anxiety. This study aims to quantify the depression levels among family members with nyaope users in Tshwane, South Africa. The study used a quantitative cross-sectional design to collect data in nine Tshwane communities. The patient health questionnaire-9 (PHQ-9) screening tool and demographic data collection questionnaires were used to collect data from a sample of 390 male and female family members who included mothers, fathers, grandparents, aunts, uncles, partners, and siblings of nyaope users, and who share a home with them. The ages of the participants ranged from 18 to 87 years, with a mean age of 47 years, while the ages of the nyaope users ranged from 17 to 55 years, with a mean age of 30 years. Depression scores ranged from 0 to 27 with a mean of 7. Depressive symptoms, as measured by the PHQ-9 scores of 5 and above, were reported by 49% of the sample. The levels of depression symptoms ranged from mild to severe, and the severity was higher among female, unemployed, and single participants. As with many others, these participants were not diagnosed and therefore were not treated. The study, therefore, identified that living with nyaope users is associated with the development of different levels of depression symptoms and has resulted in reduced quality of life among family members. The study recommends interventions that intentionally focus on families who live with individuals who are addicted to nyaope. Those interventions should focus on screening and treatment of depression and other mental disorders.

## 1. Introduction

Substance abuse is a major and growing devastating problem in South Africa with just more than 13% of the population having used a substance during their lifetime [1]. In South Africa, there is limited data on the prevalence of drug use, [2], despite the significant increase in drug use since 1994, when South Africa emerged as the most attractive largest market for illicit drugs in sub-Saharan Africa. This increasing prevalence of drug use can be partially explained by the rapidly changing social and economic climate, increased demand, availability and promotion of drugs [2,3]. In the midst of this, a relatively new cocktail psychoactive drug, known as nyaope, has flooded the drug market in South Africa. The drug emerged and became popular between 2000 and 2006, particularly in the Tshwane townships of Mamelodi, Soshanguve and Atteridgeville. It is a very addictive cocktail of drugs and is commonly used in poor Black townships in various provinces [4].

When a family has a member who uses illegal substances, the family may experience emotional and psychological stresses and the distress partly emerges from the increased responsibilities that the family is subjected to, and their limited ability to cope with the caring responsibilities [5,6,7]. These findings are supported by a recent study by [8], which indicates that, indeed, families of substance users are placed under several stressful challenges, such as family conflict, significant financial pressures, and psychological illness. The substance abuse problem is even more difficult and burdensome when dealing with adolescents [8,9]. In some extreme cases, family members even feel the need for legal protection from the person abusing substances. The negative social impact of substance abuse is often also felt by neighbours, friends, and co-workers, as it interferes with trust and the abuse of the relationships, and substance users are likely to find themselves increasingly isolated from their families or communities [10,11,12].

The literature reports on family members of substance abusers, who do not use substances themselves, expressing symptoms of depression, anxiety, and negative feelings [13]. Psychological problems may include sleeping difficulties, headaches, fear, low self-esteem, guilt, and anger. The degree of poor psychological health correlates with the impact that the substance user has on the family. A significant association was observed between substance user disorders in the patients’ spouses and children as well as in their families, where the most frequent disorder noted was depression (40.5%), followed by generalised anxiety disorder (21%), minor interpersonal and children’s behavioural problems at 15% [14,15]. In addition, financial strains, the disruption of domestic routine, constraints to social and leisure time, physical violence, and damage to property present challenges to the families [16]. Furthermore, families also deal with a stigma that is commonly directed at people with mental illness [17].

There is indeed a substantial link between mental disorders and substance use disorders. Mental, neurological, and substance use disorders have previously been reported to cause an estimated 13% of the total global burden of disease. Mental disorders affect all age groups and the impact is commonly experienced in low- and middle-income countries, resulting in a significant increase in the burden of non-communicable diseases [18,19]. The main contributory factors to mental disorders include not only individual attributes, such as the lack of skills to manage one’s thoughts, emotions, behaviours, and interactions with others, but also unsupportive social, cultural, economic, political and environmental factors, such as national policies, poor social protection, low living standards, working conditions, and a lack of community social support [20,21,22].

Depression and substance abuse often co-occur, and externalising problems seems to be a significant driving mechanism for depression [23]. Depression alone accounts for 4.3% of the global burden of disease and is among the largest single causes of disability worldwide (11% of the global population is disabled), particularly for women [24,25,26]; additionally, substance abuse contributes to negative cognitive changes [27] and interrupts normal brain maturation if used during adolescence [28]. Moreover, both depression and anxiety have been identified as common mental disorders among both adults and children [19,26], and manifest as other physical conditions, where they are associated with considerable morbidity, more severe pain, greater disability, and a poorer quality of life [28]. Common mental disorders remain largely undetected and untreated in primary healthcare settings [29]. Both depression and anxiety have a huge economic impact, both in the form of treatment costs and days lost in productive labour [30,31,32,33]. It can therefore be argued that substance abuse not only affect the user, but also thepeople that relate to the user. Although nyaope is a South African phenomenon, there is limited data on its impact on the mental health of families and communities, hence the purpose of this study is to assess the prevalence of depression symptoms among family members with a nyaope user, who live in various communities in the City of Tshwane, South Africa.

## 2. Materials and Methods

### 2.1. Study Design

The study was quantitative and cross-sectional. The data were collected from a sample of family members who were living with a nyaope user in the City of Tshwane communities, South Africa.

### 2.2. Study Population

The study population consisted of family members who could be partners, mothers, fathers, grandparents, aunts, uncles, and siblings, who share a home with the nyaope users. Due to cultural and socio-economic situations, among Black South Africans, family extends beyond the definition of the nuclear family, and it is common for people to live with various members of the extended family. The participant needed to be over the age of eighteen years and live in any of the nine City of Tshwane communities, namely, Mamelodi, Soshanguve, Mabopane, Ga-Rankuwa, Olivenhoutbosch, Atteridgeville, Winterveldt, Pretoria North, and Hammanskraal, which predominantly consists of Black people.

### 2.3. Study Setting, Sampling, and Sample Size

Participants were recruited using two methods: (1). local non-governmental organisations (NGOs) that provide a range of services to nyaope users from the Tshwane Metros, and (2). the snowball technique, which entails asking family members who have participated in the study to inform the research team about other families who have a relative who uses nyaope. Using the Raosoft sample size calculator with an estimate of 20,000 nyaope users in Tshwane and estimating a family member for each nyaope user, using a 5% margin of error, confidence interval level of 95%, and 50% response distribution, a minimum sample size of 377 was calculated.

### 2.4. Study Inclusion and Exclusion Criteria

The participants were male or female partners, mothers, fathers, grandparents, aunts, uncles, and siblings of nyaope users with whom they have shared a home for at least a year, were aged 18 years or older, and who could provide informed consent in the study. The minimum period of nyaope use was from one year. The exclusion criteria are families of users who may use other drugs but not nyaope, and families with nyaope users that have been using it for less than a year.

### 2.5. Data Collection Tools

The research team collected data using the demographic data collection questionnaire and Patient Health Questionnaire (PHQ-9) screening tools. Interviews with participants were conducted at their homes. Participants were given a brief explanation about the study and allowed to ask questions or seek clarifications regarding the study, and, when ready, they were requested to provide informed consent by signing the informed consent form.

### 2.6. Data Analysis

The raw quantitative data from 390 participants were entered in Microsoft Excel, cleaned, and coded. The data was imported to the STATA version 14 (StataCorp., College Station, TX, USA). The demographic data were analysed descriptively, and shown as the means, proportions, and percentages. The 9-item version of the PHQ-9 scale range from 0 to 27, since each of the 9 items can be scored from 0 (not at all) to 3 (nearly every day). The total scores from the PHQ-9 tool were used to determine the prevalence of depression symptoms. Scores of 0 to 4 were classified as having no depression, and for those with depression symptoms, they were classified as mild (5–9), moderate (10–14), and severe (15–27). The recommended cut-off for the PHQ-9 severity index is a score of 9. Anyone who scores 10 or above can be considered to be suffering from clinically significant symptoms of depression. Linear regression was used to explore the association of demographic variables with the PHQ-9 scores, using a *p*-value of 0.05.

### 2.7. Ethical Considerations

An ethical clearance certificate was granted by Sefako Makgatho Health Sciences University Research Ethics Committee (SMUREC/H/270/2019: PG). All participants provided written informed consent to participate in the study. Confidentiality of the participants was maintained by using participant IDs, and not their names, in the research report.

## 3. Results

### 3.1. Characteristics of the Participants

The final study sample size was 390, which was slightly higher than the minimum estimated sample size of 377. The participants were primarily parents (42%, *n* = 165), followed by siblings (35%, *n* = 137), and the majority were females (78%, *n* = 304). Their age ranged from 18 to 87 years with a mean age of 47 years. Most of the participants were single (56%, *n* = 218), and 92% (*n* = 360) were Christians. The majority (62%, *n* = 242) had a secondary qualification as their highest level of education, while *n* = 14 (4%) did not have any formal education. Unemployment was high at 46% (*n* = 180). Language comparisons showed that Setswana was the most commonly used (33%, *n* = 130), followed by Sepedi (31%, *n* = 120). Soshanguve and Ga-Rankuwa had the highest number of users (*n* = 97, 25%, and *n* = 94, 24%, respectively). Table 1 depicts the demographic profiling of the participants.

### 3.2. Characteristics of the Nyaope Users

The users were predominantly male (93%, *n* = 363) and single (73%, *n* = 285). Their ages ranged from 17 to 55 years, where the mean age of the users was 30 years. A high number of users have secondary qualifications as their highest level of education (87%, *n* = 341), and the majority of the users (92%, *n* = 357) were unemployed. Table 2 shows the demographic characteristics of nyaope users.

The duration of nyaope use ranged from 1 to 21 years, where the mean duration of nyaope use was 9 years. Ga-Rankuwa, Hammanskraal, Soshanguve, and Mamelodi had the majority of the users having used nyaope for more than 10 years, which supports the reports that nyaope use emerged from these townships, as presented in Table 3 below.

**Figure 1:**
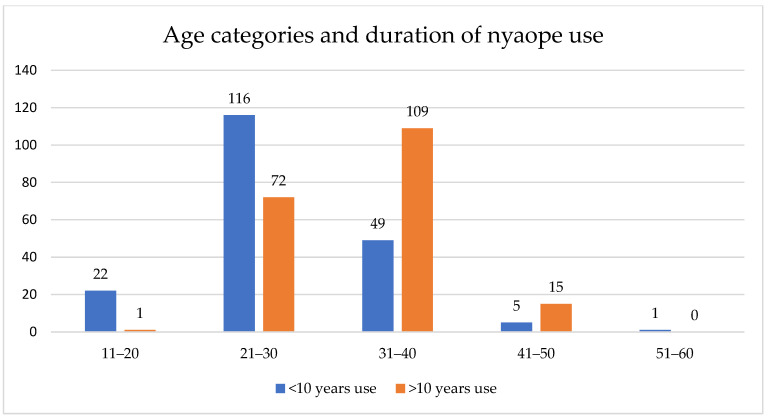
Duration of use by the age groups of the users.

The older age group reported nyaope use for a longer period, many of them for more than 10 years, as shown in Figure 1.

The majority (87%, *n* = 339) were living with their families, the majority (61%, *n* = 237) were admitted to a rehabilitation centre at some stage, and almost half (49%, *n* = 193) have been arrested.

### 3.3. Prevalence of Depression Symptoms

The PHQ scores ranged from 0 to 27, with a mean of 7 and a standard deviation of 6. The results of the depression categories are shown in Table 4. Of the 390 participants screened, 48.5% (*n* = 189) had PHQ scores of more than 4, which implies that they tested positive for depression symptoms from the results of the scale. The depressive symptoms ranged from mild to severe, as shown in Table 4.

Furthermore, almost half (45%, *n* = 177) of the sample reported that they did not find it difficult to perform their work, take care of things at home, or get along with other people, while 2% (*n* = 9) found it extremely difficult. On the other hand, 41% (*n* = 163) found it somewhat difficult and 11% (*n* = 41) stated that they found it very difficult to perform their work, take care of things at home, or get along with other people.

### 3.4. Factors Associated with Depression

Linear regression analysis was conducted on all the socio-demographic variables to explore their association with the development of depression symptoms, using a *p*-value of 0.05. The variables that were found to be significantly associated with the development of depression symptoms were the age of the participant (*p*-value = 0.034), religion of the participant (*p*-value = 0.029), relationship with the nyaope user (*p*-value = 0.017), marital status of the participant (*p*-value = 0.007), and the highest education level of the user (*p*-value = 0.028)

Although not statistically significant, the gender of the participant (*p*-value = 0.082) and number of times that the nyaope user was admitted for rehabilitation (*p*-value = 0.068) were strongly associated with depressive symptoms.

The place of residence, language, highest level of education and employment status of the participant were not significantly associated with the development of depressive symptoms, Additionally, living-at-home status and whether the user had been arrested were found not to be significantly associated with the development of depression symptoms (Table 5).

### 3.5. Multiple Regression–Depression Symptoms

Multiple regression analysis was conducted to predict the depression symptoms from age of participant, religion of participant, relationship with nyaope user, marital status of participant, and highest education of user. The religion of participant (*p* = 0.03), marital status of the participant (*p* = 0.01), and highest education level of the user (*p* = 0.02) were significant predictors of depressive symptoms (*p* < 0.05), shown in Table 6.

## 4. Discussion

The study aimed to quantify the levels of depression symptoms among family members living with a nyaope user in Tshwane communities. Although the family is historically referred to as a group of two or more people related by marriage or adoption, birth, and lineage, who live together and share financial resources regularly [34,35], this definition continues to change, and a growing variety of human attachments are being included under the definition of family. For many societies, including the communities in which the current study was conducted, family is considered to include people who provide the primary source of attachment, nurturing, and socialisation [35], and who need support if one of them uses harmful substances. These changing dynamics of the family also imply that substance abuse interferes with a wider circle of people, whose mental health is likely to be negatively affected by substance abuse [36,37]. Most family members who participated in this study were older, between the ages of 50–60 years, with a majority of them being Christians, having a secondary qualification as their highest level of education. Almost half of the interviewed family members were unemployed, with Setswana being the most dominating language, as this study was conducted in Tshwane, which predominantly consists of Tswana-speaking people. The sample consisted of a high proportion of female participants, which confirms previous findings that women are more likely to participate in studies than men [14,31]. Most of the participants who were reported to be the most distressed if their children used substances were parents, mostly mothers [38]. This could be because mothers are perceived to be overprotective and nurturing by nature.

Contrary to other studies that found that the employment of parents is one of the contributing factors that lead nyaope use, as most adolescents are left at home alone and unsupervisedthis study found that most parents were unemployed and at home full-time, available to supervise their children; however, their children are addicted to nyaope [38,39]. The high proportion of single mothers who participated in the study is a confirmation of absent fathers in many South African families [37,38], which increases the difficulties of raising children, and the outcomes expose the gap left by the absent fathers [40]. Such children are also at an increased risk of being left without supervision, a phenomenon of ‘latchkey children’, which has been reported to increase the likelihood of children engaging in undesirable behaviours, such as experimenting with nyaope or other substances. This situation also compromises their educational success [39].

As with other nyaope studies, the vast majority of the users (92%, *n* = 357) were single and unemployed, and this aligns with their lack of a life plan and purpose, due to the nyaope addiction. Unemployed people show a constant decrease in overall life satisfaction [7], and the ambition needed to achieve a better life is often suppressed. Moreover, while being unemployed is a risk factor for substance abuse, substance abuse is a risk factor for losing a job and finding it difficult to secure a job [41]. A majority of users were between the ages of 21–30 years, which is a critical age group where they should be finishing school, beginning to look for employment, and taking on other responsibilities as young adults; however, this study highlights that most of these users only have a secondary qualification, which places them at a disadvantage when it comes to employment. The long period of nyaope use, as well as the advanced age of some of the users, confirms that, once started, it is difficult to stop nyaope, and many of those who started as adolescents continue the behaviour into adulthood. Many of the users have not accessed rehabilitation services, and this could be explained by the inadequate services available, the discouragement because of the high relapse rates, and the normalisation and acceptance of their situation because of the high number of users [4].

The study found that almost half (49%) of the sample presented mild to severe depression symptoms. The levels of depression symptoms ranged from mild to severe, and this could be attributed to the sociodemographic composition of the participants. As these results may not be used as a clinical diagnosis, the easy use of this screening tool enables the identification of high-risk participants that need further interventions. The study shows that the high levels of depression symptoms and the severity are higher among female, unemployed, and single participants. The linear regression analysis found a statistically significant association between the development of depressive symptoms and age, religion, the relationship with the nyaope user, marital status, and highest level of education. Furthermore, the study found that religion, marital status, and highest education level were significant predictors of depressive symptoms. The results in this study show a significant association between age and depression symptoms, which indicates that older participants are more likely to develop depressive symptoms, which is supported by a study by [42]. The data seem to suggest that the less educated the person is, the more likely they will use nyaope; this was also observed in another study [43]. The results in this study show a significant association between age and depression symptoms, which seem to suggest that older participants are more likely to develop depressive symptoms [42]. Sharing a home with a nyaope user is strongly associated with the development of depression symptoms. This is also confirmed by previous research [35]. The number of times the user is admitted for rehabilitation has also been identified as one of the variables that may trigger depression symptoms.

The higher prevalence of depressive symptoms, as well as the increased severity of symptoms among women, are aligned with previous findings that women are at an increased risk of depression [43,44], particularly if the depression is associated with the use of psychoactive substances of their children [9]. The literature seems to suggest that hormonal changes might be the main contributing factor to this high prevalence of depression in women [45]. Additionally, a negative parenting style has been reported to be associated with depression [46,47]. Moreover, the high unemployment and unfavourable social environment of the research setting contributes to and increases the risks of nyaope use, [39,48,49] of which the parents and families have little control. Because unemployment robs people of their dignity, it may contribute to the high prevalence, as well as the severity of depression among the participants. The high unemployment rate observed in this study aligns with the country’s high unemployment rate particularly among Black people.

## 5. Conclusions

The scourge of nyaope use in South Africa, especially in the Tshwane areas, has been well documented. While the literature reports that mental health can be affected by the use of substances by immediate family members, this study is the first to quantify the depressive symptoms among family members of nyaope users. The study identified important differences in the depressive symptoms between the family members of nyaope users and indicates that sociodemographic characteristics are related to levels of distress. It is noted that using the PHQ-9 screening test does not constitute a clinical diagnosis of depression, however it enables the identification of high-risk participants. A more robust diagnosis is therefore required to confirm these findings and to initiate further interventions. The challenges related to nyaope and its extreme behavioural components, its physical, social, and mental manifestations, are not limited to the user, but present real challenges to the family and the communities. This requires a change in how nyaope use is viewed as it can no longer be ignored; moreover, these findings warrant a further in-depth understanding of the contribution of socio-demographic factors to mental distress, which may promote greater insight into the nature of the problem in this population, and point to ways of more effective interventions.

### 5.1. Recommendations

It is recommended that interventions for mental health services be developed so that they are available to family members and other members of the community. Such services can be in the form of screening for depression and anxiety, as well as lay counselling services whose purpose is to prevent depression, screen to identify those who are depressed, as well as refer those that need active treatment for depression associated with nyaope use by their family members. Furthermore, education and awareness campaigns on the prevention of substance abuse and mental disorders for communities, including schools and churches, must be intensified. A collaboration between NGOs, schools, churches, and communities, together with health professionals and the government, is recommended in addressing this increasing use of nyaope and the dealers. Difficulties accessing rehabilitation centres, high levels of relapses after rehabilitation, and the high cost of substance abuse treatment call for the government to create more effective, efficient, and affordable rehabilitation centres. Skills development programmes and employment opportunities could also help in eradicating the abuse of nyaope, as most of the users are unemployed and unemployment plays arole in the abuse of substances. The researchers in this study are of the view that more research studies are needed to gain a greater understanding of the challenges faced by families living with a nyaope user.

### 5.2. Limitations of the Study

The study design is cross-sectional, which provides only a snapshot of the depressive symptoms at a specific point in time, rather than over a prolonged period. The inclusion criteria included the prerequisite that the family member of the participant should be a nyaope user, whose symptoms are easy to observe. However, the concurrent use of other substances was not excluded.

## Figures and Tables

**Table 1:** Demographic profiling of families with a nyaope user.

Variable	*N*	%
Relationship to nyaope user		
Parents	165	42
Siblings	137	35
Other (aunts, uncles, and cousins)	55	14
Grandparents	33	9
	390	100
Gender		
Female	304	78
Male	86	22
	390	100
Age Categories		
11–20	16	4
21–30	63	16
31–40	74	19
41–50	56	14
51–60	84	22
61–70	61	16
71–80	28	7.2
81–90	7	1.8
	389	100
Marital Status		
Single	218	56
Married	99	25
Widowed	60	15
Divorced	13	4
	390	100
Highest level of education obtained		
Secondary	242	62
Primary	80	21
Tertiary	52	13
None	14	3.5
Missing data	2	0.5
	390	100
Employment status		
Unemployed	180	46
Employed	104	27
Pensioner	95	24
Student	10	2.7
Missing data	1	0.3
	390	100
Language		
Setswana Sepedi	130 120	33 31
isiZulu	48	12
Xitsonga	27	7
isiNdebele	23	6
Sesotho	21	5.4
isiXhosa	7	2
Tshivenda	4	1
siSwati	3	0.8
Missing data	3	0.8
Afrikaans	2	0.5
English	2	0.5
	390	100
Location of the participants and nyaope users		
Soshanguve	97	25
Ga-Rankuwa	94	24
Mamelodi	80	21
Hammanskraal	75	19
Winterveldt	21	5
Atteridgeville	9	2
Pretoria North	6	2
Mabopane	5	1
Olievenhoutbosch	3	0.7
	390	100

**Table 2:** Demographic profiling of the nyaope user.

Variable	*N*	%
Gender		
Male	363	93
Female	27	7
	390	100
Age category		
21–30	188	48.2
31–40	158	40.5
11–20	23	5.9
41–50	20	5.1
51–60	1	0.3
	390	100
Marital status		
Single	285	73
Married	5	27
	390	100
Highest level of education obtained		
Secondary	341	87.4
Primary	32	8.2
Tertiary	14	3.6
Missing data	3	0.8
	390	100
Employment status		
Unemployed	357	92
Employed	29	7
Student	4	1
	390	100

**Table 3:** Places of residence.

	<10 Years’ Use	>10 Years’ Use	Total
Ga-Rankuwa	65	29	94
Hammanskraal	48	27	75
Soshanguve	33	64	97
Mamelodi	29	51	80
Winterveldt	6	15	21
Atteridgeville	5	4	9
Mabopane	3	2	5
Pretoria North Olivenhoutbosch	3 1	3 2	6 3
	*n* = 193	*n* = 197	*n* = 390

**Table 4:** Screening results for depression symptoms using PHQ-9.

PHQ-9 Ranges	Classification	*n*	%
0–4	None	201	51
5–9	Mild	83	21
10–19	Moderate	65	17
20–29	Severe	41	11
		390	100

**Table 5:** Factors associated with the development of depression symptoms.

Variable	*p*-Value
Participant-related variables	
Area/place of residence/location	0.44
Age of participant	0.03 *
Gender of participant	0.08
Language of participant	0.83
Religion of participant	0.03 *
Relationship with nyaope user	0.02 *
Marital status of the participant	0.01 *
Highest Education of the participant	0.52
Employment status of the participant	0.76
User-related variables	
Age of user	0.10
Gender of user	0.27
Number of years using nyaope	0.58
Marital status of the user	0.69
Highest level of education of the user	0.03 *
Employment status of the user	0.756
Number of times admitted for rehabilitation	0.07
Lives at home	0.13
Number of times arrested	0.62

* Significant at *p* value of 0.05.

**Table 6:** Results of multiple regression of the socio-demographics and depressive symptoms.

Variable	*p*-Value
Participant-related variables	
Age of the participant	0.18
Gender of the participant	0.15
Religion of the participant	0.03 *
Relationship with nyaope user	0.1
Marital status of the participant	0.01 **
User-related variables	
Age of user	0.12
Highest level of education of the user	0.02 *
Number of times admitted for rehabilitation	0.51
Lives at home	0.16

* Significant at *p* value of 0.05. ** Significant at *p*-value of 0.01.

## Data Availability

The data presented in this study are available on request from the corresponding author. The data are not publicly available due to them containing information that can compromise the participants’ privacy.
